# Regulating polysaccharide structure and bioactivity via free radical degradation: a review of mechanisms, effects, and application prospects

**DOI:** 10.3389/fnut.2026.1725700

**Published:** 2026-02-13

**Authors:** Jieming Li, Shuaiyi Lv, Yulong Hu, Yuanfang Kong, Juntao Cai, Guanglei Nan, Shiqing Jiang, Shaohua Yang, Chunhong Dong

**Affiliations:** 1 Henan University of Chinese Medicine, Zhengzhou, China; 2 Henan Key Laboratory of Chinese Medicine for Polysaccharides and Drugs Research, Zhengzhou, China; 3 The First Affiliated Hospital of Henan University of Chinese Medicine, Zhengzhou, China; 4 Xinxiang Tuoxin Pharmaceutical Co., Ltd., Xinxiang, China

**Keywords:** biological activity, degradation, free radicals, polysaccharide, structure-activity relationship

## Abstract

Natural polysaccharides from food sources (e.g., ginseng, seaweed, apricot, larch) have diverse bioactivities (antioxidant, immunomodulatory, hypoglycemic) that are closely related to human nutritional health, but their structural heterogeneity (e.g., molecular weight, monosaccharide composition) impedes clear structure-activity relationship (SAR) establishment. Free radical degradation, a mild method preserving labile groups (e.g., sulfate esters), is an effective solution. This review summarizes its role in regulating polysaccharide SAR: focusing on hydroxyl radical (⋅OH)-mediated mechanisms (via Fenton-like reactions: processes that generate hydroxyl radicals through the reaction of H_2_O_2_ with metal ions/ascorbic acid), a three-stage kinetic model (rapid depolymerization, main chain scission, slow degradation), and site-specific modifications. Key structural changes (molecular weight reduction, functional group exposure/transformation, monosaccharide composition alteration, conformational shifts) are analyzed, and their synergistic enhancement of bioactivities (antioxidant, immunomodulatory, etc.) is elaborated. Such as, reduced molecular weight improves solubility, while exposed sulfate groups strengthen target binding. The optimal molecular weight range (10–1,000 kDa) and its dependence on polysaccharide sources/activity types are identified. Current challenges (degradation controllability, product characterization) and future directions (advanced techniques like High Performance Liquid Chromatography-Mass Spectrometry (HPLC-MS), Nuclear magnetic resonance (NMR), smart degradation systems) are discussed. This review provides guidance for precise preparation of functional polysaccharides.

## 1 Introduction

Polysaccharides from edible sources (e.g., ginseng, Gracilaria lemaneiformis, apricot, larch) are ubiquitously present in daily diets and exhibit a broad spectrum of biological properties closely linked to nutritional health, including immunomodulatory, antioxidant, hypoglycemic, and lipid-lowering effects (1–8). These properties originate from their structural similarities to human glycans (9, 10) and make them core functional ingredients for functional foods (e.g., hypoglycemic health foods, immunomodulatory supplements) (11–17). For example, degraded apricot polysaccharides with optimized molecular weight (5–8 kDa) have been applied in functional beverages to enhance α-glucosidase inhibitory activity (18), while radical-degraded larch arabinogalactan improves immunomodulatory effects in fermented dairy products (19). Their inherent biocompatibility, biodegradability, and low toxicity further underscore their potential in research and development (20, 21).

However, the pronounced structural heterogeneity of natural polysaccharides, encompassing variations in the molecular weight (Mw), monosaccharide composition, glycosidic linkage patterns, branching degree, and functional group modifications, represents a major obstacle in establishing clear SAR (22, 23). To overcome this challenge, degradation strategies have been employed to simplify polysaccharide structures and facilitate SAR studies (24). The current methods include physical, chemical, and enzymatic degradation (25–27). These methods differ substantially in mechanism, product characteristics, and applicability. Physical degradation (e.g., ultrasound, microwave) relies on mechanical force to break glycosidic bonds, but it often causes uneven Mw distribution. For example, ultrasound-degraded larch polysaccharides show Mw/Mn = 3.8, compared with 1.7 for radical degradation (19). Chemical degradation (e.g., acid hydrolysis) is effective, yet it leads to the loss of labile groups (e.g., sulfate groups in Gracilaria polysaccharides) (28). Enzymatic degradation (e.g., pectinase) is specific, but it is limited by enzyme cost and narrow substrate scope (e.g., galactan backbones) (29). In contrast, free radical degradation balances controllability, group retention, and cost-effectiveness, making it ideal for SAR studies of sulfated or carboxylated polysaccharides. Among these, free-radical-based degradation has garnered significant attention owing to its mild reaction conditions, high efficiency, and unique capacity to retain labile functional groups (e.g., sulfate esters) that are often cleaved under harsh chemical or enzymatic conditions (30, 31).

Recently, considerable progress has been made in understanding the effects of free radical degradation on polysaccharides and the underlying mechanisms (32–36). This review summarizes the advances in this field, focusing on degradation mechanisms, resultant structural changes, including reductions in Mw, alterations in functional groups and monosaccharide profiles, modifications in chain conformation, and the subsequent influence of these changes on biological activity. Finally, we discuss the current limitations and future directions for the development and application of free radical degradation in polysaccharide research.

## 2 Mechanism and characteristics of free radical degradation of polysaccharides

Degradation is primarily initiated by hydroxyl radicals (⋅OH), typically generated via Fenton-like reactions (e.g., between H_2_O_2_ and ascorbic acid or metal ions) (37). These highly reactive radicals attack the polysaccharide chains through hydrogen abstraction, predominantly by cleaving the glycosidic bonds (38, 39). This process is generally considered random, because each glycosidic bond has a similar probability of cleavage (40–42). A proposed three-stage model effectively describes the degradation kinetics and Mw evolution as follows (43): Stage I (Rapid Depolymerization): ⋅OH attack and cleave glycosidic bonds in the main chain, leading to a sharp decrease in molecular weight. This initial rapid depolymerization is facilitated by the cleavage of weaker intermolecular or intramolecular hydrogen bonds, which increases polymer accessibility (44). Stage II (Main Chain Scission): The cleavage of the main chain exposes additional sites for radical attack, resulting in a continued reduction in the chain length and polydispersity. Stage III (Slow Degradation): This process decelerates as shorter chain fragments and oligosaccharides become predominant. Chain-end cleavage and secondary reactions result in a gradual decline in Mw (45).

Although radical attacks are random in principle, the inherent structural diversity of polysaccharides introduces site-specific preferences. The reactivity of a glycosidic bond is influenced by the constituent monosaccharides and the degree of steric hindrance (43). For instance, linkages between galacturonic acid and neutral sugars (e.g., GalA-Rha and GalA-Xyl) reveal greater resistance to cleavage than homogalacturonan (GalA-GalA) regions (46). Similarly, in β-D-xylan, the C6 position is a favored site for radical attack (47). Recent studies on various polysaccharides further illustrated these preferences, showing that radicals often target the most abundant monosaccharides or glycosidic bonds with lower energy barriers (Table 1).

**TABLE 1:** The main characteristics of free radical degradation of different polysaccharides.

Source	Major monosaccharide composition	Primary attack site	References
*Gracilaria lemaneiformis*	Glc, Gal	Gal	(106)
Seaweed	Fuc, GlcA	Glc, Xyl, GalA	(46)
Citrus Pectin	Gal, NS	GalA	(43)
Ginseng	Glc, GlcA	Glc	(56)
*Codium cylindricum*	Man, Gal	ManGal	(91)
Fucoidan	Gal, Fuc	Gal	(107)
*Nymphaea hybrid*	Gal, GalA	Backbone: GalA Branch: Man	(72)
Fucoidan	Gal, Fuc	Backbone:Fuc Branch:Glc, GlcA	(108)

NS, Neutral Sugars; Glc, Glucose; Gal, Galactose; Fuc, Fucose; Man, Mannose; Xyl, Xylose; GlcA, Glucuronic Acid; GalA, Galacturonic Acid.

**FIGURE 1:**
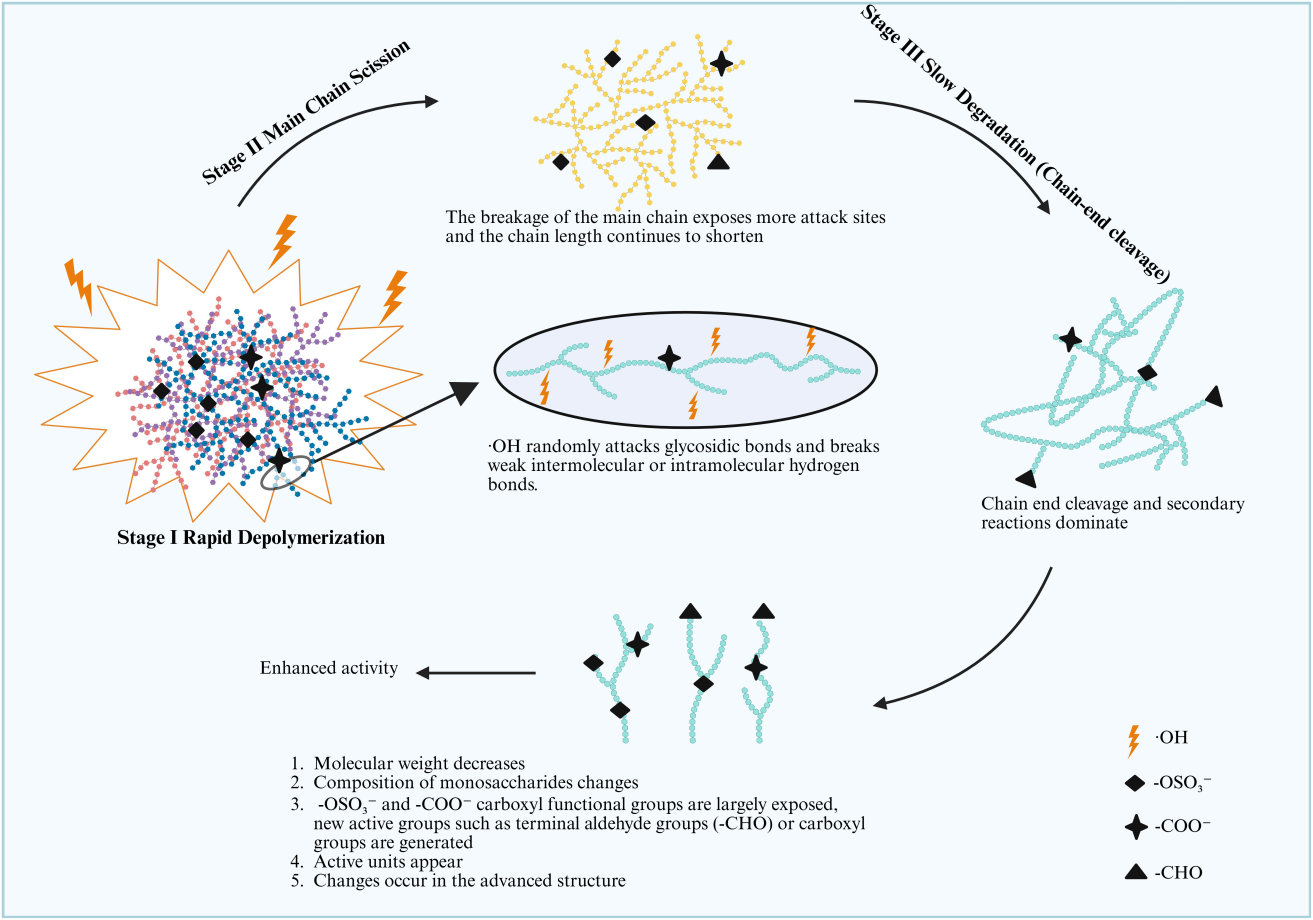
Effect of free radical degradation on polysaccharides. Active units refer to specific structural domains in polysaccharides that directly mediate biological effects. These include: (1) oligosaccharide fragments containing bioactive functional groups [e.g., sulfate esters (-OSO_3_^–^) and aldehyde groups (-CHO)]; (2) repeating sequences with specific glycosidic linkages [e.g., β-(1→3) or α-(1→4) bonds] that bind to biological targets; and (3) partially unwound triple-helix fragments exposed during degradation. These units are either newly generated or uncovered by free radical cleavage, and they are the core drivers of the synergistic enhancement of bioactivities (e.g., antioxidant, immunomodulatory) observed in degraded polysaccharides. This figure illustrates the three-stage kinetic process of polysaccharide degradation mediated by free radicals and the consequent structural changes that enhance bioactivity. In Stage I (Rapid Depolymerization), hydroxyl radicals (⋅OH) randomly attack glycosidic bonds and break weak intermolecular/intramolecular hydrogen bonds, causing a sharp decrease in molecular weight. Stage II (Main Chain Scission) follows, where the breakage of the main chain exposes more attack sites, continuously shortening the chain length. In Stage III (Slow Degradation), chain-end cleavage and secondary reactions become dominant, leading to a gradual decline in molecular weight. During these degradation stages, polysaccharides undergo multiple structural alterations: reduced molecular weight, changed monosaccharide composition, exposure or generation of active functional groups [e.g., sulfate ester (-OSO_3_^–^), carboxyl (-COO^–^), and aldehyde (-CHO) groups], and modifications in higher-order structures. These synergistic structural changes collectively contribute to the enhanced biological activity of degraded polysaccharides.

Concurrent with chain scission, the degradation process alters the functional group profile. Carboxyl groups can be converted into lactones, and new carbonyl or aldehyde groups can be introduced (48–50). Crucially, under mild free radical conditions, sulfate ester groups (-OSO_3_^–^) are typically retained as their loss primarily occurs through the removal of small sulfated oligosaccharides during dialysis (45, 51). The overall changes in the Mw, polydispersity, and functional group exposure collectively contributed to the modified bioactivities observed in the degraded polysaccharides as shown in Figure 1.

## 3 Structure-activity relationship of polysaccharides

Free radical degradation of polysaccharides induces a cascade of interconnected structural and physicochemical alterations. Although a reduction in the Mw is the most prominent change, it is intrinsically linked to other modifications. Cleavage of specific glycosidic bonds can alter the monosaccharide profile and reduce the degree of branching. Disruption of the native architecture can expose previously buried functional groups (e.g., -COOH and -OH) and modify the conformation of the polysaccharide (45). Furthermore, the decrease in Mw and chain length directly reduced the viscosity and enhanced the solubility. The synergy of these coordinated changes in Mw, monosaccharide composition, functional group accessibility, and higher-order structure ultimately mediates the observed enhancement in biological activities (52–55).

### 3.1 Molecular weight change-mediated alterations in biological activity

Degradation significantly alters the physicochemical properties of polysaccharides, with changes in the Mw being the most immediate and prominent. Polysaccharides with a Mw range of 10–1,000 kDa generally exhibit enhanced bioactivity, whereas those with Mw < 10 kDa or > 1,000 kDa often show reduced efficacy (40). Notably, this optimal range is strongly dependent on polysaccharide sources and target activities: For ginseng polysaccharides (immunomodulatory activity), the optimal Mw is 70–150 kDa (native 135 kDa shows stronger α-glucosidase inhibition than degraded 77.8 kDa) (56); for seaweed sulfated polysaccharides (anticoagulant activity), the range narrows to 3–8 kDa (57); and for apricot polysaccharides (hypoglycemic activity), 5–8 kDa is preferred (18). This dependence arises from source-specific structural motifs (e.g., ginseng’s β-(1→6) glucan branches vs. seaweed’s sulfate-rich backbones) and activity-related target binding (e.g., thrombin prefers low-Mw sulfated fragments, while macrophage receptors require larger flexible chains) (19).

High Mw (e.g., > 1000 kDa) can hinder polysaccharide diffusion in food matrices and intestinal cellular uptake, reducing their nutritional bioavailability in human diets (58). For instance, native larch arabinogalactan (Mw > 1,000 kDa) shows limited absorption in the small intestine, while degraded fractions (10–150 kDa) exhibit 2.3-fold higher bioavailability in rat models (19). In contrast, very low Mw (e.g., < 10 kDa) may compromise structural integrity, weakening nutritional functions like hypoglycemic activity (18), degraded apricot polysaccharides below 5 kDa show a 30% reduction in α-glucosidase inhibitory activity compared to the 5–8 kDa fraction, whereas very low Mw (e.g., < 10 kDa) may compromise the structural integrity and conformational stability, which are essential for bioactivity (59). Xu et al. (26) found that in obese mice fed a high-fat diet, degraded low-molecular-weight polysaccharides exhibited stronger lipid-lowering and antioxidant activities than their native high-molecular-weight counterparts. Similarly, Yan et al. (60) demonstrated that radically degraded polysaccharides having lower Mws possess enhanced free radical-scavenging capacity. Conversely, Wang et al. (56) reported that the native ginseng polysaccharide (135 kDa) exhibited stronger inhibitory activity against α-glucosidase and α-amylase than its degraded derivative (77.8 kDa).

Furthermore, native high-Mw polysaccharides often exhibit high viscosities and low solubilities. This was attributed to stronger intramolecular hydrogen bonding, which promoted the formation of dense spherical conformations (61). This compact structure reduces the solubility and masks functional groups, limiting their availability for biological interactions and absorption (62, 63). In addition, uronic acid residues within polysaccharides interact with ions and entrap endogenous molecules (38, 64, 65), thereby impeding the dissociation and release of minerals and other bioactive compounds. Degradation disrupts these compact structures, reduces their Mw, and enhances their solubility, thereby facilitating their biological activity (66, 67). A representative example is a polysaccharide from the sclerotium of Pleurotus tuber-regium, which exhibits immunomodulatory and antitumor activities. However, poor solubility results in low bioavailability. Degradation reduces Mw, thereby improving solubility and enhancing bioactivity (68). Free radical degradation is widely used to efficiently reduce the Mw of polysaccharides. For instance, Zhi et al. (69) prepared low-molecular-weight heparin via free radical degradation and demonstrated that this method can rapidly reduce the molecular weight and yield polysaccharides with targeted low-Mw profiles.

Free radical degradation not only decreases Mw but also enhances solubility, thereby potentiating biological activity (38). Moreover, a reduction in the molecular size increases the specific surface area and leads to a more open conformation, which exposes previously hidden active sites and promotes interactions with biological targets (56). Additionally, low-molecular-weight polysaccharides possess a large number of reducing and non-reducing ends, which can enhance their bioactivities (70). For example, terminal β-1,4-D-mannose residues may bind to mannose receptors on immune cells and elicit immunomodulatory effects (71).

However, its excessive degradation can be detrimental. Tan et al. (72) observed that a degraded polysaccharide from Nymphaea hybrid (46 kDa) exhibited superior free radical scavenging activity compared to a more degraded variant (16 kDa). This suggests that an overly reduced Mw may disrupt essential structural motifs and hydrogen bond-stabilized conformations, leading to diminished activity (73). Thus, an optimal Mw range exists in which the benefits of improved solubility and exposed active sites are balanced against the need to maintain structural coherence.

In addition to altering bioactivity, free radical degradation improves functional properties, including reduced viscosity, enhanced solubility, and the removal of pigment impurities, thereby increasing product purity (46). This also narrows the Mw distribution, resulting in a more homogeneous product with lower dispersity (74). Furthermore, degradation can modulate properties, such as viscosity, gelation capacity, water retention, and thermal stability (75, 76). These modifications have significantly expanded the potential applications of polysaccharides in the pharmaceutical, nutraceutical, and materials sciences fields (77). Notably, Mw reduction is not an isolated change, it synergistically promotes the exposure of functional groups. For example, degraded apricot polysaccharides show a 2.8-fold increase in carboxyl group content, as the breakdown of compact spherical conformations unmasks buried carboxyls (18). This Mw-driven group exposure directly enhances bioactivity, as seen in the stronger α-glucosidase inhibitory activity of the degraded product.

### 3.2 Change of group mediates alteration in biological activity

Free radical degradation can induce the exposure, formation, and transformation of functional groups within polysaccharides that are essential for modulating their biological activities. Studies have shown that during degradation, exposed uronic acid residues may be converted into lactones, resulting in an apparent decrease in uronic acid content (49). Wu et al. (48) observed a gradual increase in the intensity of the carboxylate-related absorption band of a degraded polysaccharide, corroborating this transformation. Recently, new functional groups have also been introduced. For instance, Huang et al. (50) identified a new absorption peak at 1730 cm^–^ in a degraded polysaccharide; this was attributed to the stretching vibration of an aldehyde group (-CHO), indicating the introduction of this new moiety (78). Furthermore, free radical degradation typically does not cleave sulfate ester groups (-OSO_3_^–^), a key advantage for retaining nutritional functions of sulfated polysaccharides in food processing. Ge et al. (51) demonstrated that this method retained > 95% sulfate groups in Gracilaria lemaneiformis polysaccharides, which is critical for their anticoagulant and lipid-lowering effects in functional seafood products. This group retention enables free radical degradation to be applied in functional food production, such as optimizing sulfated seaweed polysaccharides for blood lipid regulation. Structural evidence further confirms sulfate retention: ^1^H-NMR spectra of ⋅OH-degraded *Gracilaria lemaneiformis* polysaccharides show characteristic peaks of sulfate protons at δ 4.2–4.6 (consistent with native polysaccharides), whereas acid-hydrolyzed samples lose these peaks (45). FTIR analysis also supports this: the absorption band at 1250 cm^–^ (S = O stretching of sulfate esters) remains unchanged after radical degradation but disappears in acid-treated samples (79, 80).

In summary, free radical degradation can lead to the full exposure of active groups (e.g., sulfate esters and carboxyl groups) and may facilitate the formation of new groups (e.g., aldehydes and carbonyls) (81, 82). Modification of these functional groups directly influences the bioactivity through specific biochemical mechanisms. Antioxidant activity is closely associated with aldehyde and sulfate ester groups but negatively correlated with the degree of esterification (83–85). The mechanism is based on the properties of these groups: the negative charge of sulfate esters can effectively neutralize and scavenge positively charged free radicals (e.g., ⋅OH) (81), while the aldehyde group possesses a highly active C-H bond. The excellent electron-donating capacity (reducing power) of the aldehyde group enables direct reduction of free radicals, thereby terminating oxidative chain reactions (50). Consequently, polysaccharides with high contents of aldehyde and sulfate ester groups generally exhibit enhanced antioxidant activities.

The anticoagulant and antitumor activities are profoundly influenced by the degree of sulfation (53, 86). The negative charge of sulfate esters enables electrostatic interactions with positively charged amino acid residues on proteins, such as thrombin. This interaction inhibits thrombin activity, thereby enhancing the anticoagulant effects (87). Similarly, these electrostatic forces can strengthen the binding of sulfated polysaccharides to cancer cell surfaces. For instance, the degradation of porphyran reduced its Mw and increased the relative proportion of sulfate groups, and the resulting product demonstrated strong anti-proliferative activity against SGC-7901 and 95D cancer cells, which was attributed to enhanced sulfate group-mediated interactions (88).

Hypoglycemic and hypolipidemic activities are also strongly linked to specific functional groups. The degree of esterification is closely correlated with the inhibitory activities against α-amylase and α-glucosidase (48). Glucuronic acid residues exposed after degradation may also play a significant role in enhancing the anticoagulant activity through analogous charge-based interactions (87). Moreover, alterations in functional group content can modulate the binding of polysaccharides to biomolecules. For example, the degradation of apricot polysaccharides significantly increases the content of hydroxyl, carbonyl, and carboxyl groups. These polar groups promote binding to cholic acid, thereby regulating lipid metabolism and markedly increasing the lipid-lowering activity of the polysaccharides (89). Similarly, increased sulfate content in degraded Ganoderma lucidum polysaccharides enhances their bioactivity, making them promising candidates for treating hyperlipidemia (26).

### 3.3 Changes in monosaccharide composition mediated alterations in biological activity

Free radical degradation not only alters the Mw and functional groups, but also significantly modifies the monosaccharide composition of polysaccharides, a factor critically linked to their biological functions. These compositional shifts, including changes in molar ratios and selective removal of specific sugars, directly influence bioactivity by altering the molecular motifs available for interaction with biological targets. For example, polysaccharides rich in specific molar ratios of galactose, arabinose, and mannose are often associated with enhanced immunomodulatory activity. Arabinogalactan, a notable functional domain characterized by high arabinose and galactose contents, is a prime example of a structure that elicits a strong immune response (90). Similarly, a high prevalence of monosaccharides, such as galactose, arabinose, and glucose, is a common feature of polysaccharides that exhibit hypoglycemic effects (91). The antitumor activity of degraded pectin is fundamentally dependent on its core structural elements, primarily consisting of α-1,4-linked galactopyranuronic acid and α-1,2-linked rhamnopyranose residues (92).

Although some reports suggest that free radical degradation primarily alters the proportional ratio of monosaccharides without changing the types present, others indicate that specific sugars can be selectively removed. For example, Zhao et al. (93) observed that rhamnose was undetectable in certain degraded fractions (CSP-3 and CSP-4) of corn silk polysaccharides, and Wu et al. (48) reported the removal of fucose during degradation. This selective removal can be attributed to several factors, including the inherent susceptibility of different glycosidic linkages to radical attack, the uneven distribution of monosaccharides between the backbone and side chains, and variations in the degradation rates of individual sugar residues (50). Smaller oligosaccharide fragments generated by the cleavage of these susceptible sugars were subsequently lost during dialysis, thereby altering the final monosaccharide profile of the purified product (72). Consequently, the strategic application of free radical degradation serves as a powerful tool for obtaining polysaccharide fractions with refined sugar composition. By enhancing the relative abundance of bioactive motifs (e.g., by increasing the arabinose-to-galactose ratio for immunostimulation) or removing inert or masking sugars, degradation can potently amplify specific biological activities. A comprehensive summary of the changes in the monosaccharide composition and Mw of various polysaccharides following radical degradation is provided in Table 2. These changes in monosaccharide composition further modulate higher-order conformations: For larch arabinogalactan, ⋅OH-mediated cleavage of α-(1→3) arabinose side chains (reducing arabinose/galactose ratio from 1:9.06 to 1:31.36) weakens intramolecular hydrogen bonding, shifting the conformation from a compact sphere (average particle size 337.90 nm) to a semi-flexible coil (182.87 nm). This conformational shift enhances binding to macrophage Toll-like receptor 4 (TLR4) receptors, leading to 150% higher tumor necrosis factor-α (TNF-α) secretion, directly linking monosaccharide composition changes to conformational and activity enhancements (19).

**TABLE 2:** Changes in monosaccharide composition and molecular weight before and after degradation of polysaccharides from different sources.

Source	Monosaccharide composition									Mw (kDa)	References
	Gal	Glc	Man	Ara	GalA	Xyl	Rha	GlcA	Fuc		
*Grateloupia filicina*	96.3%	0.6%	0.2%	\	\	1.4%	0.4%	0.5%	0.5%	2 093.4	(95)
	96.2%	1.4%	0.5%	\	\	\	0.5%	1.4%	\	40.8	
	96.3%	1.5%	0.8%	\	\	\	0.3%	1.1%	\	22.6	
	90.0%	5.2%	1.3%	\	\	\	\	4.0%	\	5.1	
	92.2%	3.8%	0.6%	\	\	\	\	3.3%	\	3.0	
*Porphyra yezoensis*	87.5%	10.3%	1.5%	\	\	Trace	Trace	\	\	452.0	(109)
	91.1%	5.8%	1.3%	\	\	1.5%	0.4%	\	\	10.8	
	95.9%	1.3%	1.0%	\	\	0.1%	0.5%	\	\	10.7	
	95.5%	1.5%	1.2%	\	\	0.2%	0.5%	\	\	18.7	
	98.8%	0.4%	0.4%	\	\	0.4%	0.3%	\	\	35.5	
*Panax notoginseng*	13.8%	24.8%	5.4%	27.3%	\	20.8%	\	4.7%	3.3%	64.7	(52)
	16.5%	29.3%	8.1%	23.8%	\	20.3%	\	2.0%	\	8.9	
	19.8%	21.7%	8.8%	25.4%	\	22.3%	\	2.0%	\	6.0	
	16.4%	27.9%	\	24.8%	\	22.1%	\	8.8%	\	5.9	
*Okra*	46.2%	\	8.4%	4.6%	20.7%	\	20.1%	Trace	\	284.0	(48)
	44.5%	\	8.9%	3.1%	22.7%	\	20.8%	Trace	\	190.0	
	43.0%	\	8.5%	3.0%	24.3%	\	21.3%	Trace	\	82.7	
	42.3%	\	8.9%	2.4%	23.7%	\	22.2%	Trace	\	46.5	
*Tremella fuciformis*	1.8%	6.0%	59.5%	\	0.6%	1.7%	\	14.9%	\	814.0	(100)
	0.6%	5.6%	59.6%	\	0.6%	1.7%	\	16.3%	\	123.0	
	1.0%	5.1%	65.5%	\	0.5%	1.6%	\	11.7%	\	65.6	
	1.4%	5.0%	65.0%	\	0.5%	1.6%	\	13.0%	\	12.7	
*Grateloupia livida*	81.5%	11.0%	\	0.4%	\	1.0%	0.4%	2.6%	3.1%	23 044.3	(55)
	86.1%	6.1%	\	0.8%	\	1.0%	0.4%	2.5%	3.2%	158.5	
	87.1%	5.6%	\	0.3%	\	1.3%	0.5%	1.8%	3.5%	8.06	
Ginseng	0.4%	98.8%	0.2%	0.1%	0.4%	\	\	0.1%	0.1%	135.0	(56)
	0.5%	95.9%	1.4%	0.1%	1.2%	\	\	0.2%	0.7%	77.8	

### 3.4 Changes in structure mediate alterations in biological activity

Alterations in functional groups, monosaccharide composition, and Mw induced by free radical degradation collectively perturb the intricate network of intramolecular hydrogen bonding and charge distribution. These perturbations ultimately lead to significant changes in the higher-order spatial structures of polysaccharides, which are crucial determinants of their biological activities (94). For instance, the antitumor activity of polysaccharides is intimately linked to their stereochemical configuration, with specific structural motifs such as β-(1→3) and β-(1→6) glycosidic bonds in the repeating unit being particularly critical for efficac (95–97).

The triple-helix conformation is a classic example of a structure-activity relationship, often exhibiting superior bioactivity compared to single-stranded or random-coil conformations (98). This enhanced activity is attributed to the greater rigidity of the helix and its more efficient recognition and binding by specific receptors on immune cells. However, a triple helix is not a prerequisite for bioactivity; many potent heteropolysaccharides, which are less likely to form rigid structures, also display significant immunomodulatory effects (28). The effect of degradation on this conformation is nuanced. Tan et al. (72) demonstrated that degrading a polysaccharide that initially lacks a triple helix does not induce its formation as both the native and degraded forms exhibit similar random-coil structures. Conversely, Wang et al. (56) showed that degrading a polysaccharide with a native triple-helix structure does not completely destroy it but can cause a degree of disintegration or loosening of the helical coil. This partial unraveling is a key mechanism as it can expose previously buried functional groups and active sites, thereby enhancing accessibility to biological targets and increasing activity, even as the Mw decreases.

Beyond the overall conformation, specific structural domains are critical for function. For example, the rhamnogalacturonan I (RG-I) domain, which is characterized by rhamnose and galacturonic acid, is known to elicit a strong immune response owing to its distinct three-dimensional architecture (99). The structure of the side chains also plays a pivotal role, and the degree of branching is a key factor influencing the immunomodulatory activity of pectin polysaccharides (90). Li et al. (100) observed that free radical degradation reduced both the Mw and degree of branching, which subsequently improved solubility, altered conformation, and enhanced the interaction between polysaccharides and cells, leading to increased immune activity (101). However, the relationship between the degree of branching and immunomodulatory activity is not linear, indicating an optimal structural range rather than a simple “more-is-better” correlation (102). Because of the complexity of polysaccharide structures, the precise relationship between their spatial architecture and biological activity remains a rich area for further investigation.

## 4 Discussion and conclusion

Polysaccharides are a class of biomolecules with diverse biological roles, boasting broad application potential in food processing, cosmetics, and clinical therapeutics (103). Unlocking this potential hinge on a deep understanding of their structure-activity relationships (SAR). This review differs from broad-spectrum reviews that merely catalog degradation methods, it highlights the unique value of hydroxyl radical (⋅OH)-mediated degradation by clarifying its direct link to polysaccharide SAR: ⋅OH degradation excels at preserving labile functional groups (e.g., 95% sulfate retention in sulfated seaweed polysaccharides, confirmed by ^1^H-NMR signals at δ 4.2–4.6) (45), and enabling targeted structural modifications (molecular weight reduction, exposure or creation of functional groups like sulfate esters, aldehydes, and carboxylates, refinement of monosaccharide composition, and alterations to higher-order structures such as partial unwinding of triple helices). These structural changes are not incidental, they modulate polysaccharide interactions with cellular receptors and proteins via electrostatic forces and hydrogen bonds, directly enhancing bioactivities including antioxidant, immunomodulatory, anticoagulant, and antitumor effects. To advance the field beyond current focus on overall efficacy evaluation, several critical, concrete challenges must be addressed with more sophisticated approaches. First, high-throughput sequencing of complex oligosaccharides remains a bottleneck, even with advanced techniques like HILIC (Hydrophilic interaction liquid chromatography)-UPLC-Orbitrap-MS/MS, sequencing oligosaccharides with glucuronoarabinogalactan motifs is hindered by branch structure fragmentation interfering with sequence accuracy. For instance, distinguishing RGP-type (Rha-GalA-Pent) and GRP-type (GalA-Rha-Pent) sequences in degraded Amomum tsao-ko polysaccharides relies on specific fragment ions (m/z 300 for Pent-ABEE, m/z 476 for GalA-Pent-ABEE), a method that cannot be generalized to more complex branched structures (104). Second, industrial-scale ⋅OH degradation suffers from poor reproducibility: batch-to-batch molecular weight variation reaches 12–15%, driven by uneven ⋅OH generation and raw material batch differences, limiting its application in pharmaceutical development. Third, SAR understanding remains shallow—most studies only correlate bulk properties with activity, lacking insights into the molecular mechanisms by which specific degraded fragments interact with biological targets (105).

Against this backdrop, future research should prioritize three focused directions. First, leverage AI and molecular docking to predict ⋅OH cleavage sites, reducing experimental redundancy. Second, develop microfluidic-based smart reactors to enable real-time adjustment of reaction parameters (e.g., pH, H_2_O_2_ concentration), improving batch uniformity. Third, explore combined degradation strategies, such as ultrasound pretreatment to loosen polysaccharide triple helices without damaging primary structures, followed by ⋅OH degradation to retain active functional groups and partial triple-helical conformations, and finally enzymatic hydrolysis for precise modification of low-molecular-weight products, balancing efficiency and retention of active structural domains. Additionally, advancing the field requires in-depth analysis of degradation characteristics via integrated advanced techniques: HPLC-MS (e.g., HILIC-UPLC-Orbitrap-MS/MS) for oligosaccharide sequencing (104), NMR (^1^H, ^13^C, HSQC) for functional group verification (e.g., δ 4.2–4.6 for sulfate protons, δ 175.6 for carboxyl carbons in degraded seaweed polysaccharides), and Gel Permeation Chromatography-Eighteen Angles Laser Light Scattering (GPC-MALLS) for molecular weight distribution quantification (e.g., Mw/Mn = 1.7 for UV/H_2_O_2_-degraded Sargassum fusiforme polysaccharides indicating uniform degradation) (45). Enhancing reaction controllability and reproducibility also demands smart systems, such as, controlling plasma discharge time at 200 s yields apricot polysaccharide fragments with targeted Mw (5–8 kDa) and enhanced α-glucosidase inhibitory activity (18), alongside intelligently controlled platforms like photocatalytic reactions or combined enzymatic-chemical methods.

This review offers actionable strategies bridging basic SAR research with industrial practice for both food researchers and the functional food industry. For sulfated polysaccharides (e.g., seaweed-derived), controlling reaction pH at 5.0–6.0 minimizes desulfation, preserving anticoagulant activity for use in low-sodium functional sauces. For hypoglycemic functional foods (e.g., apricot polysaccharide beverages), targeting Mw of 5–8 kDa via plasma-assisted ⋅OH degradation enhances α-glucosidase inhibitory activity, meeting the nutritional needs of type 2 diabetes patients (18). In food processing, degraded larch arabinogalactan (10–150 kDa) boosts yogurt’s immunomodulatory activity by 150% without altering texture or shelf life. In structural analysis, combining HPLC-MS (oligosaccharide sequencing) and NMR (functional group verification) avoids misinterpreting degradation product compositions (45).

In summary, this review’s core contribution lies in illuminating the direct connection between ⋅OH-mediated degradation and polysaccharide SAR, emphasizing ⋅OH’s distinct advantages in preserving labile functional groups and enabling targeted modifications that synergistically enhance bioactivities. While critical challenges (complex oligosaccharide sequencing, industrial reproducibility, shallow SAR understanding) persist, the proposed future research directions and practical industrial strategies lay a solid foundation for advancing the development of functional polysaccharides in nutraceuticals, functional foods, and food processing.
